# Tracking invasion events: phylogeography of *Hyalomma marginatum* in the Mediterranean basin with a focus on Southern France

**DOI:** 10.1186/s13071-025-06927-4

**Published:** 2025-10-14

**Authors:** Carla Giupponi, Hélène Jourdan-Pineau, Célia Bernard, Valeria Blanda, Maria Bourquia, David Bru, Oscar Cabezón, Laura Carrera-Faja, Johan Espunyes, Yuval Gottlieb, Charlotte Joly-Kukla, Laurence Malandrin, Noureddine Mechouk, Andrei Daniel Mihalca, Thomas Pollet, Phonsiri Saengram, Alessandra Torina, Félix Valcárcel, Zati Vatansever, Laurence Vial, Abderrahmane Zahri, Hélène Verheyden, Karine Huber

**Affiliations:** 1 Centre de Coopération Internationale en Recherche Agronomique Pour le Développement (CIRAD), UMR ASTRE, Montpellier, France; 2 ASTRE, Univ Montpellier, CIRAD, INRAE, Montpellier, France; 3 Istituto Zooprofilattico Sperimentale Della Sicilia, Palermo, Italy; 4 Unité Parasitologie et Maladies Parasitaires, Institut Agronomique et Vétérinaire Hassan II, Rabat, Morocco; 5 Wildlife Conservation Medicine Research Group (WildCoM), Departament de Medicina i Cirurgia Animals, Universitat Autònoma de Barcelona (UAB), Bellaterra, Spain; 6 Unitat Mixta d’Investigació IRTA-UAB en Sanitat Animal, Centre de Recerca en Sanitat Animal (CReSA), Campus de la Universitat Autònoma de Barcelona (UAB), Bellaterra, Spain; 7 IRTA, Programa de Sanitat Animal, Centre de Recerca en Sanitat Animal (CReSA), Campus de la Universitat Autònoma de Barcelona (UAB), Bellaterra, Spain; 8 Koret School of Veterinary Medicine, The Hebrew University of Jerusalem, Rehovot, Israel; 9 ANSES, INRAE, Ecole Nationale Vétérinaire D’Alfort, UMR BIPAR, Laboratoire de Santé Animale, 94700 Maison-Alfort, France; 10 Oniris, INRAE, BIOEPAR, Nantes, France; 11 Department of Parasitology and Parasitic Diseases, University of Agricultural Sciences and Veterinary Medicine of Cluj-Napoca, Cluj-Napoca, Romania; 12 Grupo de Parasitología Animal, Animalario del Departamento de Reproducción Animal, INIA-CSIC, Madrid, Spain; 13 Department of Parasitology, Faculty of Veterinary Medicine, Kafkas University, Kars, Turkey; 14 Université de Toulouse, INRAE, CEFS, Castanet-Tolosan, France

**Keywords:** *Hyalommamarginatum*, Tick, Phylogeography, France, Mediterranean basin, Genetic diversity, Biological invasion

## Abstract

**Background:**

*Hyalomma marginatum* is a hard tick vector of various pathogens, including Crimean-Congo Hemorrhagic fever virus, recently detected in French specimens. This species has a wide distribution from North Africa to Eastern Europe and has only recently been considered established in Southern France. These changes in species distribution led us to explore the genetic structure of tick populations in the Mediterranean basin and attempt to infer the origin of French populations.

**Methods:**

We used two mitochondrial markers (12S rRNA and Cytochrome Oxidase 1) and genotyped ticks from nine Mediterranean countries. We compared genetic indices and haplotypic composition between these countries and the various French geographical populations.

**Results:**

Across all countries, we showed significant genetic differentiation, with a certain proximity between neighboring countries. We found very different genetic compositions among the French geographic populations: some exhibited signs of recent expansion, while others suggested the presence of ancient populations.

**Conclusions:**

It is possible that small populations of *H. marginatum* were already present in France and are now more abundant. This recent change in population structure could be owing to increased human activity and climate change. These factors, combined with a potentially high level of phenotypic plasticity, could facilitate *H. marginatum* conquest of more northerly latitudes in France and other European countries.

**Graphical Abstract:**

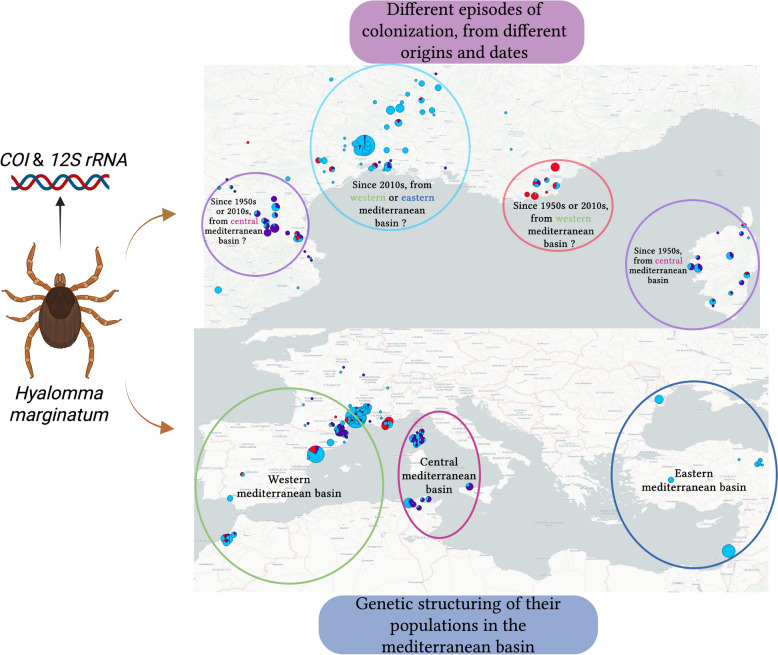

**Supplementary Information:**

The online version contains supplementary material available at 10.1186/s13071-025-06927-4.

## Background

Among the Ixodidae, species of the genus *Hyalomma* are vectors of numerous pathogens (viruses, bacteria, protozoa), which can infect humans and animals, including *Coxiella burnetii*, *Theileria* spp., *Babesia* spp., *Anaplasma* spp. and *Rickettsia* spp. [1]. They are also vectors of an arbovirus, the Crimean-Congo Hemorrhagic fever virus (CCHFV), which is emerging in Western Europe [2, 3]. The genus *Hyalomma* encompasses 27 species and has a Eurasian and African distribution [4, 5]. One of these species, *Hyalomma marginatum*, is present in several Mediterranean countries, from the South Maghreb to the Iberian Peninsula and the East of the Mediterranean basin (Turkey, Balkans) and is progressively expanding its range towards the north and west of Europe [6]. In France, the species was documented in Corsica as early as the 1940s [7, 8] but documentation on its presence in the southern regions of mainland France remained scarce, despite a few reports of specimens in the Var and Pyrénées-Orientales departments [8, 9]. However, the scientific community considered that these were not established populations, and that they more likely reflected periodic introduction of individuals by migratory birds. At that time, continental France was the only area north of the Mediterranean considered uncolonized. Since 2015, it has been shown that populations have become established first in the Hérault and Gard departments [10], then in the Var and Pyrénées-Orientales departments [11]. This species is now established along the French Mediterranean arc and its presence is spatially aggregated, i.e. there are zones of presence separated by zones where it is absent. The range of this tick is also increasing along the Rhône valley. Recent studies have suggested that the south of France may represent a newly colonized area for *H. marginatum* for which the extent of the invasion is still unknown [12].

An invasion may be considered to have occurred when a group of individuals has been introduced into a new area, in which they have established themselves, increased in number and spread geographically [13]. Biological invasion depends on the success of several non-exclusive processes that can be combined: a change in the migration patterns of the invasive species, a change in environmental conditions increasing the number of favorable habitats, and post-colonization evolution resulting in a better adaptation of the species to the environmental conditions of the newly colonized territories [14].

While the ability to disperse is therefore essential to a biological invasion, ticks, owing to their parasitic biology, have only low dispersal capacities per se. Their dispersal is therefore highly dependent on the movements of their hosts. The more mobile the host, the greater the migration rate between populations [15]. *Hyalomma marginatum*, whose immature stages parasitize small vertebrates and especially birds, can be transported over very long distances [16]. In addition, this species is a ditropic tick, meaning that engorged larvae remain on the same host to molt and feed again as nymphs, which leaves more time for it to be transported by the host. Captures of migratory birds infested with *H. marginatum* suggest that these birds may serve as a means of transporting juvenile stages from their areas of origin [17]. Morphological identification of immature stages of genus *Hyalomma* is considered difficult [18]. However, the combined use of morphological approaches and molecular taxonomy techniques allows identification of these individuals at the species level. The horse (*Equus caballus*) which is one of the preferred hosts of this species in the adult stage, could also play a role in the dispersion of the tick [19]. The significant importation of this animal from Spain and Italy to France may have contributed to the introduction of the species within French territory. Additionally, livestock importation and exportation between countries (i.e., horses and cattle, *Bos taurus*), as well as the movement of wild ungulates (such as wild boar, *Sus scrofa*), may also contribute to the dispersal of this tick.

But the host is not the only parameter at play: the tick must be able to find an ecological niche that is favorable to its establishment. In their free-living phases (outside hosts), ticks are highly sensitive to environmental conditions. This vulnerability determines the temperature and humidity ranges that characterize their maximum spatial range. Several modeling studies have been carried out in Europe to explain and predict the distribution of *H. marginatum*. These studies indicate a preference of this tick for high temperatures, but low precipitation and relative humidity [12, 20–22]. Global changes can promote biological invasions, with climate change notably contributing to the spread of *H. marginatum*. Rising global temperatures have likely allowed this species to extend its range further north in Europe, aided by the dispersion of its hosts [23].

The aim of this work is to identify the origin of *H. marginatum* populations present on French territory by documenting the invasion and expansion underway in South of France, using phylogeographic and population genetic tools based on mitochondrial markers.

Phylogeography on mitochondrial markers has provided valuable insights into the migration patterns of tick species. This has led, for example, to the identification of two lineages and colonization events for *Rhipicephalus appendiculatus* in sub-Saharan Africa [24], or to the identification of a weak gene flow between different populations of *Ornithodoros verrucosus* [25]. In the context of biological invasions, phylogeography can be used to confirm the ongoing expansion with lower genetic diversity expected in the new environment compared with native areas. As a result, we expect to find more marked genetic differentiation between native and newly-established populations, resulting from a bottleneck followed by the effects of post-introduction genetic drift. The level of differentiation observed will also depend on the rate of migration between the area of origin and the invaded area [26]. Knowing the origin of *H. marginatum* populations present in France, and how these individuals were transported by different hosts, will enable us to better assess the risk of introducing new pathogens, and to implement control measures, notably by monitoring animal imports [27, 28]. It could also tell us to which environment it is pre-adapted and help predicting how far it could expand [29].

For our study, we analyzed two mitochondrial markers, the *cytochrome oxidase I (COI)* gene and the *12S rRNA* gene, in 699 *H. marginatum* individuals collected from nine different countries, including four populations from France. We have developed a sampling strategy that combines long-established populations, such as those in the countries of origin of this species, with more recently established populations, such as those in southern France. We also included areas where the species is not established, suggesting that it has probably been transported there very recently via their host. In addition, ticks have been collected from a wide range of hosts, both local animals and wild animals such as migratory birds. It is the combination of these different signals that will enable us to formulate hypotheses on the invasion patterns of this species.

## Methods

### Biological materials

A total of 709 *Hyalomma* ticks, morphologically identified as *H. marginatum*, were collected in nine countries covering the range of this species around the Mediterranean basin. Of these, 480 ticks were collected from 97 localities, were collected in France between 2008 and 2024 (Additional file 1). Collection campaigns were carried out in nine departments where the tick is present: Ardèche, Aude, Corse-du-Sud, Drôme, Gard, Haute-Corse, Hérault, Pyrénées-Orientales and Var. French departments are classified as NUTS 3 regions within the Nomenclature of Territorial Units for Statistics (NUTS) system of the European Union. French ticks were also sent to the lab directly or originated from the French citizen science project PiroGoTick, including 14 from areas where *H. marginatum* is not yet established. Through scientific collaborations, a collection of 229 ticks was obtained from the following countries: Italy, Spain, Israel, Algeria, Morocco, Turkey, and Romania. For Tunisia, we directly obtained sequences already produced for the genes of interest for 17 individuals [30] (OQ263353, PV175379–PV175394 (12S)/PQ885461–PQ885477 (COI)). We have also included sequences from the complete mitochondrial genomes deposited on Genbank of eleven individuals of *H. marginatum* from Turkey (NC_056189, MW366628–MW366633, MN885800, MT270686–MT270688) [31]. This brings us to a total of 737 samples in our dataset. The exchange of biological samples was conducted in full compliance with the Nagoya Protocol on Access to Genetic Resources and the Fair and Equitable Sharing of Benefits Arising from their Utilization (ABS).

Most ticks were collected from horses (*n* = 437) and cattle (*n* = 148), and, to a lesser extent from wild boar (*n* = 60), local birds (*Erithacus rubecula*, *Panurus biarmicus*, *Passer montanus*, *Turdus merula*, *n* = 27), long distance migratory birds (*Acrocephalus scirpaceus*, *Luscinia megarhynchos*, *Motacilla alba* *n* = 12), other mammals (*n* = 3), and environment (*n* = 22). Each individual was identified morphologically by using relevant key for species identification [32]. The immature stages, which are more difficult to identify, were identified morphologically by an expert acarologist. In all cases, a molecular identification step complemented morphological identification. Only ticks belonging to the *H. marginatum* species were retained for further phylogeographic analyses. Its stage, sex, host, and place and date of collection were recorded (Additional file 1).

### Amplification and sequencing of mitochondrial markers

Two mitochondrial markers commonly used for phylogeographic analyses on ticks were selected for this work, the 12S rRNA and the cytochrome oxidase I (COI) genes [15, 33, 34]. At the beginning of this study, four markers were used, including the cytochrome b and 16S rRNA genes. However, given the limited additional information provided by these markers, they were not selected. The 12S rRNA was amplified using the specific primers T1B/T2A [35], and two primer pairs were used to amplify the COI gene, TY-J-1449/C1-N-2312 and Cox1F/Cox1R [36, 37] (Table 1).

**Table 1:** Primer names, primer sequence and the edited sequence length of the amplified product used in this study

Gene	Primer name	Reference	Edited sequence length (bp)	Sequence
12S rRNA	T1B	Beati and Keirans [35]	341	5′-AAACTAGGATTAGATACCCT-3′
	T2A			5′-AATGAGAGCGACGGGCGATGT-3′
COI	TY-J-1449	Murrell et al. [36]	773	5′-AATTTACAGTTTATCGCCT-3′
	C1-N-2312			5′-CATACAATAAAGCCTAATA-3′
COI	Cox1F	Lv et al. [37]	773	5′-GGAACAATATATTTAATTTTTGG-3′
	Cox1R			5′-ATCTATCCCTACTGTAAATATATG-3′

Ticks, after having been frozen at −80 °C for at least 2 h before DNA extraction, were individually dry-ground with 0.3 mm steel balls a Tissue Lyser (Qiagen). Samples were disrupted in a frequency of 30 Hz during 3 min. Total DNA was extracted using a commercial Nucleospin Tissue column kit (Macherey–Nagel) according to the manufacturer’s instructions and eluted in a final volume of 60 μl of elution buffer. PCR amplification reactions were performed in a reaction volume of 50 μl. The PCR mix consisted of FailSafe™ PCR 2X PreMix C, the two primers (10 pmol each), FailSafe™ Taq (1.25 U) and genomic DNA (2 μl). PCR amplification conditions were as follows: for COI, an initial denaturation step at 95 °C for 5 min followed by ten cycles of 92 °C for 1 min; 42 °C for 40 s; 72 °C for 1 min 30 s; 32 cycles of 92 °C for 1 min; 46 °C; 72 °C for 1 min 30; and a final extension step at 72 °C for 7 min. For the 12S gene the conditions are slightly different: an initial denaturation step at 95 °C for 5 min followed by five cycles of 94 °C for 15 s; 51° for 30 s; 72° for 30 s, 25 cycles of 95° for 15 s; 53° for 30 s; 72° for 30 s; and a final extension step at 72 °C for 5 min. For each amplification reaction, negative controls were performed, consisting of 48 μl of PCR mix and 2 μl of deionized water. The COI gene was amplified using two different primer pairs to obtain a larger sequence size. Only one primer from each pair was used for sequencing (Cox1F and C1-N-2312) and the two sequences were then assembled using Geneious v.6.0.5 (Biomatters) assembling tool.

Gel migration was performed to ensure the quality of the amplification and to check the size of the amplified DNA fragments. If necessary, PCR products were purified on gel using the GFX PCR DNA and Gel Band Purification Kit (GEHealthcare). The PCR products were sent for sequencing by AZENTA-GENEWIZ using the SANGER method. The obtained sequences were analyzed using Geneious v.6.0.5 (Biomatters) and aligned with MUSCLE algorithm [38]. DNA sequences for *H. marginatum* were deposited in GenBank under Accession Number (PV051478–PV052132) for 12S rRNA and (PV019529–PV020183) for COI (Additional file 1).

For each tick, in addition to the morphological identification, we performed a molecular identification on the COI and 12S rRNA gene. The acquired sequences were identified at the species level using the Basic Local Alignment Search Tool (BLAST) by comparing them against published reference sequences in the GenBank database. A sequence identity of over 99% between the query sequence and the reference specimen was used to confirm sample identification.

### Genetic diversity and structure

Ticks identified as non-*H. marginatum* were excluded from further analyses, leading to 699 individuals in the dataset. Genetic diversity within and between populations and geographical areas was assessed using DnaSP 6.12.03 [39] using the following statistics: the number of haplotypes (*h*), haplotypic diversity (Hd), nucleotide diversity (*π*) and the average number of nucleotide differences (*k*). The basic geographical unit that we considered for the analyses was chosen according to the publication by Bah et al. [12], which identified distinct zones in the distribution of *H. marginatum* in the French Mediterranean basin with spatially aggregated populations of ticks. There are four geographical units, hereafter henceforth referred to as “geographical groups.” The first group consists of the departments of Aude and Pyrénées-Orientales, located in the western part of France (referred to as P-OA in the rest of this article). The second group encompasses Gard, Hérault, Ardèche, and Drôme (referred to as GHAD in the following). The third group includes the departments of Var, while the fourth comprises the island of Corsica, situated to the east.

To infer genealogical relationships between populations, we constructed a haplotype network using the median-joining network method [40] for each gene, then for all concatenated genes using NETWORK 4.6.1.2. Genetic structure was assessed using a Bayesian clustering method implemented in the RhierBAPS 1.1.3 package [41] on R, derived from the hierBAPS algorithm [42]. The analysis was performed using 100 replicate runs, with a maximum number of populations (K) set to 97, corresponding to the number of currently sampled sites.

To estimate the genetic differentiation between the populations studied, we used the F_ST_ statistic, based on the average number of differences between different sequences sampled in the same subpopulation and the average number of differences between sequences sampled in the two different subpopulations sampled. This statistic was calculated from Eq. 3 of Hudson et al. [43] for all population pairs using DnaSP software. The populations were grouped by country and for France, by “geographical group”, and compared with each other.

The influence of various environmental and ecological factors on the population groups was tested by molecular analysis of variance (AMOVA) [44, 45] using ARLEQUIN 3.5.2 software [46]. In this analysis, a population was considered when at least 5 individuals came from the same collection site. Four independent tests were carried out. For France, we tested a spatial structure of haplotypes by grouping our population by two geographical levels (two tests), French departments and geographical groups. We also tested an ecological unit, the climate. For climate, we used the LANMAP3 classification, which classifies European climates for each locality into 8 different categories [47] and has already been used to classify the environments of *H. marginatum* [48]. The last test was carried out at the Mediterranean scale, to see whether groupings by country was relevant. Stage, sex, and host parameters are overly influenced by geographical area, creating overlapping or uneven patterns. As a result, it was not possible to isolate or test the individual effects of these factors in the analysis.

### Demographic history

The genetic signature of past demographic changes was studied by country and French geographical groups using neutrality tests based on Tajima’s D statistics and Fu’s Fs with ARLEQUIN 3.5.2. Significant negative values (i.e., a significant rejection of the null hypothesis) are expected in populations that have experienced an increase in effective population size [49–51].

We also performed a mismatch distribution test using ARLEQUIN v.3.5.2. In populations that have experienced a rapid demographic expansion, the mismatch distribution is anticipated to exhibit a smooth, unimodal curve [52]. This test was carried out in populations where the number of sequences was sufficient for the model to converge and be relevant, i.e., French populations.

## Results

### Taxonomic affiliation

After analysis of COI and 12S rRNA sequences, all individuals were correctly assigned to the genus *Hyalomma,* and the vast majority allowed us to confirm that they belonged to the species *H. marginatum* (699 individuals). Fourteen individuals *Hyalomma scupense* were identified in France: four in Hérault (OM743220–OM743223), one in Bouches-du-Rhône (OM743219) and nine in Pyrénées-Orientales departments. We also found this species in Italy (two specimens) and in Spain (six specimens) (PQ899451–PQ899462/PQ894010–PQ894026). *Hyalomma lusitanicum* was detected in two countries: Spain (seven individuals) and Morocco (five individuals) (PQ900077–PQ900084/PQ902093–PQ902099). One *Hyalomma rufipes* (PQ897005/PQ899450) and two *Hyalomma excavatum* (PQ899448–PQ899449) were also found in Algeria. Additionally, one *Hyalomma excavatum* was identified in Morocco (PQ899169).

### Genetic diversity and structure

Throughout the entire dataset, the 12S gene exhibited lower polymorphism than the COI gene, with 12 haplotypes and a haplotypic diversity of 0.184, compared with 57 haplotypes and a haplotypic diversity of 0.673 for the COI gene (Additional file 2).

When individuals are grouped by population, genetic diversity varies greatly (Table 2). The first thing to note is the presence of a single haplotype for the Romanian population whereas we found up to 41 haplotypes for France. Apart from Romania, haplotype diversity ranges from 0.504 (Turkey) to 0.856 (Algeria). Within France, geographic populations show very variable haplotype diversity, ranging from 0.542 (GHAD) to 0.79 (Corsica).

**Table 2:** Genetic diversity indices for sequences analyzed on concatenated genes (12S & COI), according to geographical populations and countries

Country	Population	Ns	Nl	S	*h*	*k*	Hd	*π*	*D*	Fs
	Corsica	61	12	20	19	2.277	0.79	0.00214	−1.306	1.361
	GHAD	253	45	21	22	1.022	0.542	0.00114	−**1.511**	32.53
	P-OA	91	17	9	9	2.164	0.745	0.00234	0.664	20.67
	Var	47	9	8	8	1.706	0.605	0.00174	0.104	13.2
France		466	97	35	41	1.925	0.717	0.00154	−**1.412**	−14.901
Algeria		18	3	9	8	1.824	0.856	0.00171	−0.649	5.299
Israel		25	1	8	10	0.993	0.69	0.00102	−1.22	5.61
Italy		8	1	4	3	1.536	0.607	0.00142	−0.2	3.736
Morocco		40	4	17	17	1.876	0.807	0.00186	−**1.665**	2.907
Romania		11	1	–	1	–	–	–	–	–
Spain		80	12	10	11	1.039	0.55	0.00113	−0.737	−5.58
Tunisia		17	4	7	6	1.794	0.647	0.00162	−0.393	3.846
Turkey		34	8	9	9	0.64	0.504	0.00064	−**1.7**	−1.4

*Ns* Number of sequences, *Nl* Number of localities, *S* Polymorphic sites, *h* Number of haplotypes, *k* Average number of nucleotide differences, *Hd* Haplotypic diversity, *π* Nucleotide diversity, *D* Tajima’s D, *Fs* Fu’s Fs. For Tajima’s *D* and Fu’s Fs, significant values (*P*-value < 0.05) are highlighted in bold

**Fig. 1:**
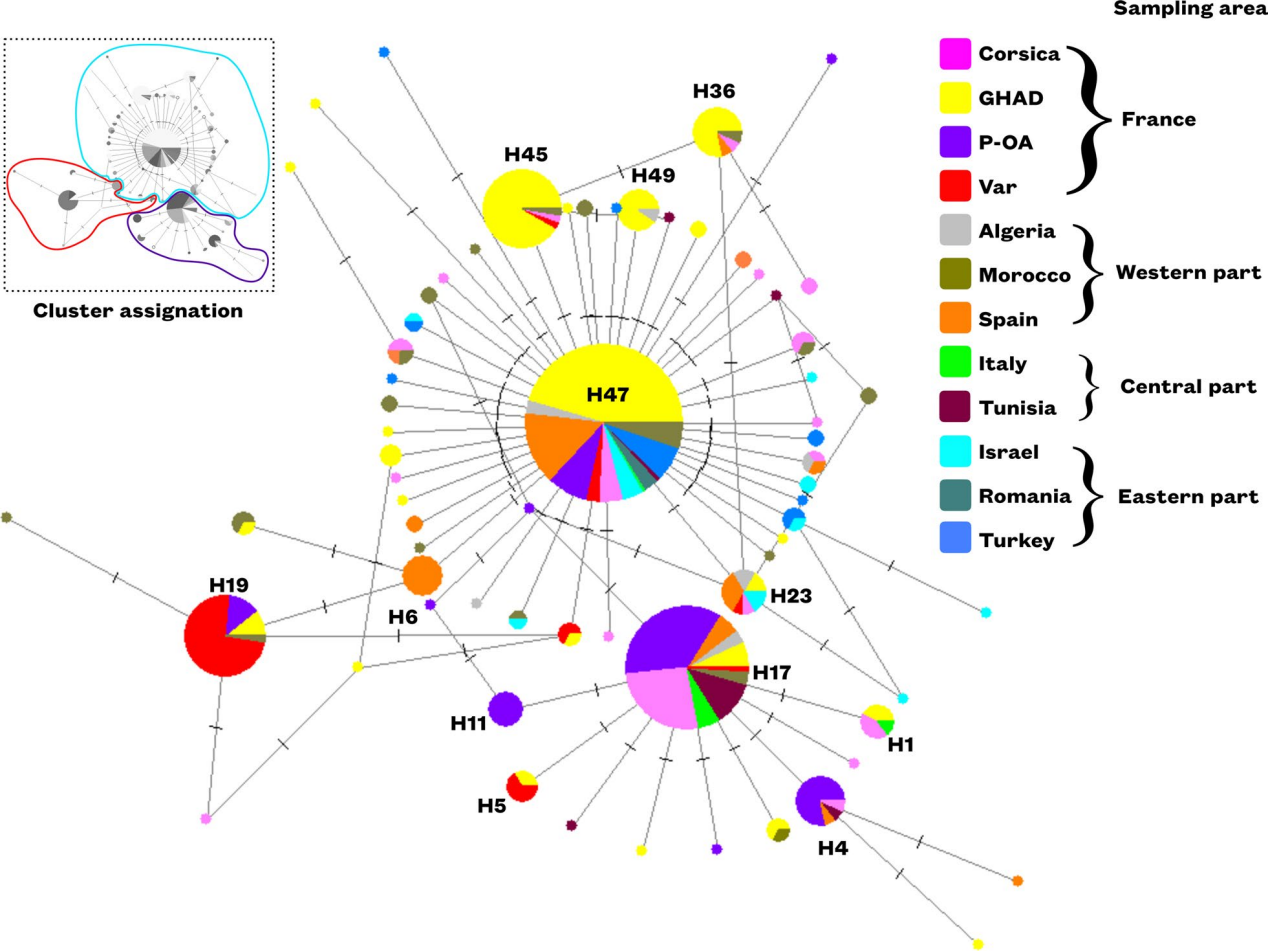
Median-joining haplotype networks for the two concatenated genes. The size of the circles is proportional to the numbers of individuals and the dot corresponds to one mutation. The colors represent the geographical areas of the individuals. The countries in the legend have been grouped by geographical proximity: eastern, central and western countries of our sampling area. Top left of figure: correspondence between haplotypes and clusters defined by hierBAPS analysis (using the same color code as for the figures below)

The haplotypic composition of each population varies greatly from one geographical area to another (Table 3). Haplotype 47 is found in all populations and predominates in most countries. It is particularly present in Spain, Israel, Turkey, Algeria, and Romania. In France, it is dominant in the GHAD population. The second most abundant haplotype is haplotype 17, mainly found in Italy, Tunisia, and in the Corsican and P-OA populations. The only population whose predominant haplotype does not include either of the two above-mentioned haplotypes is that of Var, where haplotype 19 is almost exclusive to this area. Finally, we note that some populations present numerous minor haplotypes (*n* < 5 sequences): Morocco, Israel, Turkey, and Corsica. The haplotype network (Fig. 1), based on the two concatenated genes, has a star-like structure centered around two major haplotypes: haplotype 47 and haplotype 17. Haplotype 47 is connected to a multitude of minor haplotypes, whereas haplotype 19 is linked to only two minor haplotypes. Both haplotypes 17 and 19 differ from haplotype 47 by three and two SNPs, respectively.

**Table 3:** Haplotype frequencies of concatenated genes (12S and CO1), according to geographical populations and countries

Country	Population	Ns	Hap 1	Hap 4	Hap 5	Hap 6	Hap 11	Hap 17	Hap 19	Hap 23	Hap 36	Hap 45	Hap 47	Hap 49	Minor haplotypes (*n* < 5)
	Corsica	61	0.05	0.02				0.37			0.02	0.02	0.28		0.25
	GHAD	253	0.01		0.01			0.02	0.02		0.04	0.15	0.66	0.04	0.05
	P-OA	91		0.12			0.09	0.34	0.03		0.01		0.34		0.07
	Var	47			0.09			0.02	0.63	0.02		0.02	0.22		0.00
France		466	0.01	0.03	0.01		0.02	0.13	0.08	0.01	0.03	0.08	0.49	0.02	0.09
Algeria		18						0.16		0.11			0.53	0.05	0.16
Israel		25								0.08			0.60		0.32
Italy		8	0.13					0.63					0.25		
Morocco		40						0.08	0.03		0.03	0.03	0.46		0.38
Romania		11											1.00		
Spain		80		0.01		0.13		0.06		0.04	0.01		0.66		0.09
Tunisia		17		0.06				0.59					0.18		0.18
Turkey		34											0.74		0.26
Total		699	0.01	0.02	0.01	0.01	0.01	0.13	0.06	0.02	0.02	0.06	0.52	0.01	0.12

**Fig. 2:**
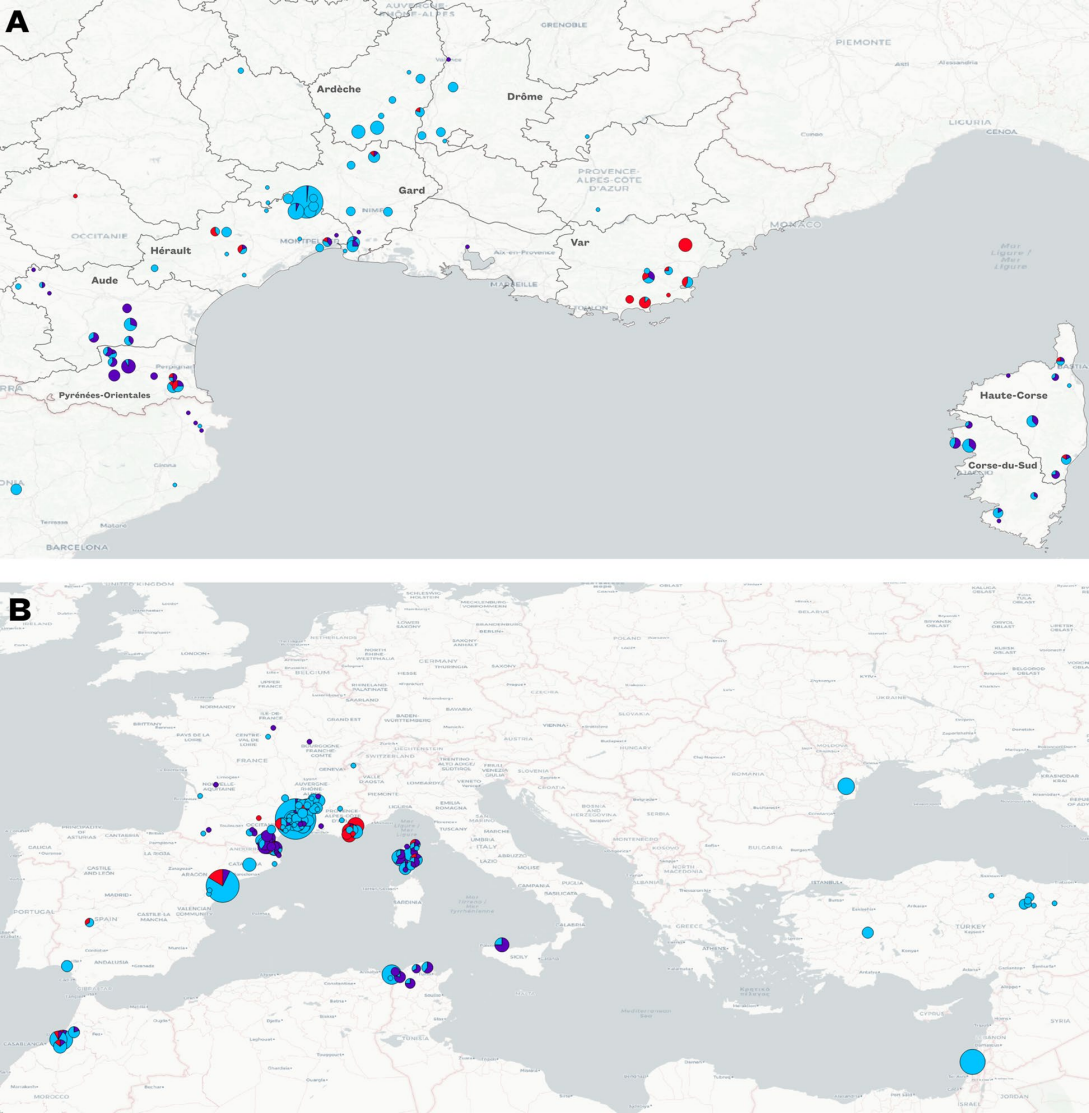
Genetic clustering by hierBAPS of *H. marginatum* populations of **A** South of France **B** Mediterranean basin. Colors represent the cluster to which individuals have been assigned (cluster 1 = violet, cluster 2 = red, cluster 3 = blue). Circle size is proportional to the number of individuals

Genetic clustering using hierBAPS gave us an optimal number of three clusters (Fig. 2). The specimens from Turkey, Israel, and Romania all belong to cluster 3. In the GHAD population, 91.1% of ticks were assigned to cluster 3. Similarly, the majority of specimens from Spain, Morocco, and Algeria are part of cluster 3 (78.5%, 79.5%, and 84.2% respectively). In contrast, individuals from Italy and Tunisia belong mainly to cluster 1 (75% and 70.5% respectively). The P-OA and Corsica population are more heterogeneous, with 56% and 44.1% of individuals belonging to cluster 1, and 37.3% and 54.2% to cluster 3, respectively. In Var, 65.9% of the individuals were assigned to cluster 2. As for individuals from French northern departments where the tick has not yet established itself (Gironde, Hautes-Alpes, Pyrénées-Atlantiques…) no clear clustering pattern emerges. All three clusters are present, with cluster 3 as the most prevalent, accounting for 57.1% of individuals.

**Fig. 3:**
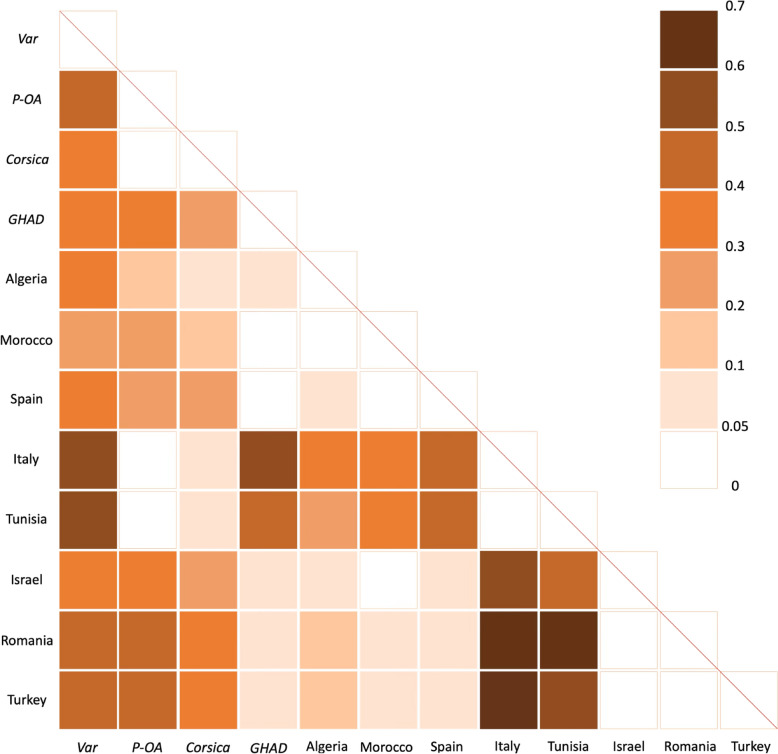
Estimated *F*_ST_ according to geographical groups compared. *F*_ST_ values range from 0 (white) to 0.7 (dark orange). French populations are indicated in italic

The genetic distance parameters values (*F*_ST_) vary widely between geographic areas, ranging from 0.001 (between Spain and Morocco) up to 0.676 (between Italy and Romania) (Fig. 3). The Var population shows a moderate differentiation from other areas, with *F*_ST_ values ranging from 0.241 (with Morocco) and 0.547 (with Italy). The population of Morocco seems less differentiated from the others, with *F*_ST_ values ranging from 0.001 (with Spain) to 0.393 (with Italy), while Italy is the most differentiated, with *F*_ST_ values ranging from 0.002 (with Tunisia) to 0.676 (with Romania). However, for the latest, it is possible that the small sample size (*n* = 8) caused *F*_ST_ to be higher than expected [48].

The four AMOVA tests (Table 4) show consistently high intra-population variations of around 60%. On the other hand, inter-group variations were much more variable. We found no significant variation linked with climate (*P*-value = 0.112) and countries (*P*-value = 0.821), but a rather high proportion of variance explained by geographic groups (31.969%, *P*-value = 0) and by departments (22.943%, *P*-value = 0). The latter are the only ones where inter-group variation is greater than inter-population variation.

**Table 4:** Molecular analysis of variance (AMOVA) of the concatenated genes (12S & COI)

	% Variation within populations	% Variation among populations within groups	% Variation among groups
Departments (France)	**54.568**	**22.488**	**22.943**
Geographical groups (France)	**49.637**	**18.392**	**31.969**
Climate classes (France)	**55.099**	**41.569**	3.331
Countries	**64.708**	**40.352**	−5.06

Intra-population variation, inter-population variation within groups, inter-group variation, of population according to different groupings for the four independent tests. Significant values (*P*-value < 0.05) are highlighted in bold

### Demographic history

Among the demographic indices, none of the Fu’s Fs tests turned out to be significant (Table 2). The Tajima’s *D* test is significant for Morocco (−1.665, *P*-value < 0.032), Turkey (−1.7, *P*-value < 0.026), France (−1.412, *P*-value < 0.039) and the French population GHAD (−1.511, *P*-value < 0.037).

**Fig. 4:**
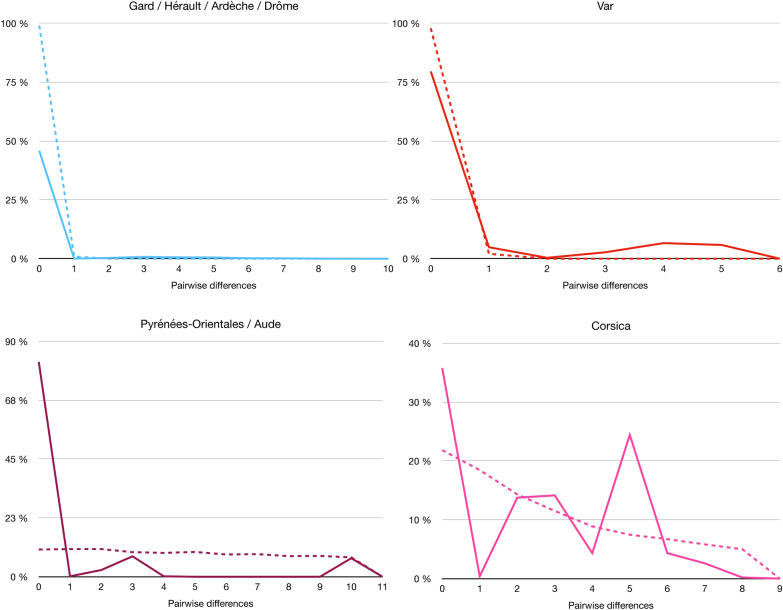
Mismatch distribution among pairwise differences among haplotypes in the four French populations. GHAD in blue, P-OA in purple, Corsica in pink, Var in red. Observed data and theoretical expected distributions are represented by solid and discontinuous line, respectively

Concerning the mismatch distribution test (Fig. 4), the distribution observed for the Var and GHAD populations shows a significant peak at 0 mismatches, although this peak is slightly less pronounced than the theoretical one. This is followed by low, fluctuating values for the other mismatch categories, with a few sporadic bumps. This low frequency of mismatches in the intermediate categories suggests that there is relatively little genetic diversity between sequence pairs, which could indicate a recent population expansion. In contrast, the other two populations have a very different pattern. The PO-A population shows a bimodal distribution with two major peaks at 4 and 110 mismatches, and the Corsican population shows a multimodal distribution with numerous scattered peaks. For the latter, the theoretical distribution looks quite flat, suggesting a rather ancient population.

## Discussion

As described in 2016 [10], *H. marginatum* is now considered established in southern France, with its distribution characterized by spatially aggregated areas of presence separated by areas of absence [12]. Mitochondrial markers provide valuable insights into the structure and origins of these populations. During biological invasion, bottleneck and genetic drift tend to reduce genetic diversity in invasive populations. Accordingly, we expected high haplotypic diversity in the native areas and in long-established populations, such as those in Corsica. On the contrary, we expect a lower diversity in newly settled populations of mainland France.

### The *Hyalomma* genus in France

Within the taxon Ixodidae, the genus *Hyalomma* is a genus that has diverged recently, within the last 50 million years ago [53], and that shows a high cryptic diversity, which could be the consequence of divergences between very recent lineages. Indeed, the *Hyalomma* genus shows high intra-specific morphological variability, interspecific hybridization [54] and the presence of cryptic species complexes [53]. Despite prior morphological identification of our samples, in addition to *H. marginatum*, we identified three other species of *Hyalomma* in our sampling: *H. scupense*, *H. lusitanicum*, and *H. rufipes*.

Specimens of *Hyalomma scupense* were found at three distinct locations in France, in the Hérault, Pyrénées-Orientales and Bouches-du-Rhône departments. In the Pyrénées-Orientales department, we seem to be dealing with a settled population, with nine individuals discovered from cattle in 2024. This seems in agreement with the reports of *H. scupense* in the Pyrenean mountains in 1959 and 1993 [8, 55]. This raises the question of whether other *Hyalomma* species, such as *H. marginatum*, could have long been established in the region but misidentified. However, this hypothesis needs to be nuanced as *H. scupense* favors bovine hosts and a more humid environment, perhaps enabling it to survive more easily than *H. marginatum* in the Pyrenean mountains.

*Hyalomma lusitanicum* is endemic to the Iberian Peninsula [56], where Crimean-Congo Hemorrhagic Fever is currently circulating [57]. Multiple individuals were found in our samples from Spain and Morocco, where they seem to live in sympatry with *H. marginatum*. Recent collections show the existence of established populations in the Bouches-du-Rhône department (Stachurski et al*.*, *in prep.*), whereas the species was no more observed in France since the 1960’s, raising the question of a recent introduction or a residual population [8].

The *Hyalomma rufipes* specimen found in Algeria was probably transported by migratory birds, such as all other *H. rufipes* specimens observed North of Sahara, in France [10], Germany [58], Sweden [59] or Netherlands [60]. A specimen was also observed in 2019 in the Aude department, France (Stachurski personal com). Hoogstraal et al. [61] already noted that *H. rufipes* nymphs could be observed in spring on several bird species migrating from Africa to Europe or Asia.

Finally, the *Hyalomma excavatum* specimens found are consistent with their known North African range [62].

### Genetic structure of *H. marginatum* in the Mediterranean basin

Taking the Mediterranean basin as a whole, we see that genetic diversity varies greatly from country to country. African populations, particularly those in Algeria and Morocco, show greater genetic diversity, consistent with the longer establishment of these populations. It could also be that these populations benefit from a more consistent gene flow, owing to the numerous migrations of birds passing through these countries within the African continent and toward Europe, but also livestock trading. On the other hand, populations in Eastern Europe and the Middle East display lower genetic diversity, with Romania showing particularly low levels. However, this must be weighed against the low number of individuals and the fact that all samples were collected in a single sampling event in one locality. Geographical and abiotic factors could explain this difference: continental climate and colder winters in the Balkans could limit dispersal and genetic mixing, increasing the isolation of populations. But it could also be a remnant of more ancient historical events. Sands et al. [53] suggest that geological and climatic events have influenced the distribution and speciation of the genus *Hyalomma*. It is possible that the Last Glacial Period, a climatic event of glacial and interglacial periods spanning 116,000 to 14,700 years ago [63], created a refuge area for *H. marginatum* in Africa where the climate remained more stable over the last few thousand years [64]. In Europe, life cycle completion may have been hindered by lower temperatures and remnant populations could have undergone bottlenecks, reducing their genetic diversity. Even today, *H. marginatum* can be found over a much wider period of the year in North African countries, such as Moroccan specimens, which we were able to collect in abundance in September.

We can also see a genetic proximity between countries in the same geographical areas: the eastern countries (Israel/Romania/Turkey), the western countries (Algeria/France/Morocco/Spain) and the “central” countries (Italy/Tunisia). This pattern further underlines the marked spatial structuring into large geographical areas and the possible gene flows within areas between geographical populations in this species.

### The origins of French populations

Our results identified three distinct genetic groups in France, each with varying degrees of diversity indices. This suggests different genetic origins for each of these populations potentially arising from several introduction events in the past, rather than a single invasion event in the 2010s. It is therefore possible that each French population has its own history of introduction.

In the Gard and Hérault departments, *H. marginatum* had only been observed very occasionally before 2015, and was completely unknown to farmers. However, it is now very abundant, particularly in equestrian facilities. The very low haplotype diversity observed in this population, the demographic tests showing signs of a recent population expansion, and the absence of any mention of this species in these departments in literature might suggest that this is a more recently imported population. In the last few years, its distribution has extended further north along the Rhone valley, reaching the Ardèche and Drôme departments, where the species is gradually becoming established [12]. The genetic proximity of this population to those of Morocco and Spain suggests a southwestern origin. The large number of Spanish horses imported into the region may explain the introduction of this tick and the genetic proximity of these populations, although migratory birds could also play a role. However, the genetic proximity of the population of Israel, Turkey, and Romania indicates that an eastern origin, via migratory routes of birds from the East, cannot be completely ruled out.

In the Var department, a population was described in the 1950s near the Massif de l’Estérel [8] and then in the 1970s [9]. Afterward, the presence of this species was no longer reported in the literature until populations of *H. marginatum* were confirmed in mainland France in 2016 [10]. When questioned, some local livestock farmers recall this tick always been present, but that its abundance has dramatically increased in the last 10 years, while others have never seen it before. The unique haplotypic composition of this department supports the hypothesis of a small, genetically isolated population that has persisted for decades. Changes in climatic conditions or in human activities that favors the presence of hosts could have allowed this population to expand and thus to be detected in recent years.

Finally, the question arises as to whether *H. marginatum* populations were present in the Pyrénées-Orientales and Aude departments at earlier dates. As stated before, Rageau’s [9] and Morel’s [8] reports of *H. excavatum* in these departments questioned the possibility of the presence of a population of *H. marginatum* in these departments that would have been misidentified. Similar to the Var department, breeders report a longstanding presence, but with a more recent increase in abundance. However, the haplotypic richness and demographic tests point to an older population that is genetically less isolated.

A recent study by Joly-Kukla et al. [65] on the spatial distribution of pathogens carried by *H. marginatum* in southern France revealed a clustered distribution of *Rickettsia aeschlimannii* (a secondary symbiont). The infection rates and bacterial loads differed significantly across these geographical clusters possibly due to the introduction of tick populations from different geographic regions, each carrying distinct microbial communities. The observed differences between ticks from Pyrénées-Orientales and Aude, compared with those from Gard and Hérault, mirrored the haplotypic divergence described in our study.

The Corsican populations are genetically very close but geographically distant to P-OA. These are the two French populations with the greatest haplotypic diversity and similar compositions, which could suggest a link between these two areas. It is possible that there is frequent genetic flow between these two populations although both populations have close genetic links with Tunisia and Sicily. This suggest they have originate from these countries, for example via North–South bird migration routes [66]. This genetic proximity between distant geographical areas raises questions about the tick’s long-distance dispersal modes. The presence of individuals in departments where the tick has not yet become established itself shows that the species is frequently imported into unoccupied areas but environmental conditions are probably not favorable to its establishment. While migratory birds play a major role in the introduction of ticks to northern Europe [66, 67], it is not certain that they are the main factor in the expansion of *H. marginatum*. Most of the individuals we found on birds, whether migratory or not, were collected in Gard or Hérault French departments and belonged to cluster 3 (i.e. haplotypes 47, 45, and 23) the common local cluster which Is also widespread in Northern Africa. This may be consistent with a hypothesis of importation of these ticks from Algeria to the Camargue region, a crossing point for migratory birds in the Gard department. Besides, it is highly probable that the import of engorged adults via equines and cattle favors a more rapid establishment [68], since one *Hyalomma* oviposition can generate an average of 7000 eggs [69] and up to 15,500 [70]. Thus, the movement of large mammals by humans could accelerate an otherwise lengthy process. Additionally, local wildlife could quickly spread the species at short distance [71, 72]. For example, a large number of nymphs were found in the Gard region on *Turdus merula*. This bird spends a lot of time on the ground and moves over shorter distances, which could allow the tick to spread from one place to another, as was observed along the Rhône valley. Moreover, the human is a means of transportation that is sometimes forgotten for *H. marginatum*. Ticks can be carried by long-distance means of transport such as buses and cars without necessarily parasitizing humans [73]. Urbanization, livestock farming, and other human activities can influence the distribution of *H. marginatum*, even in areas where it is already present, helping it to expand.

Mitochondrial DNA has enabled us to gain initial insights into the history of the *H. marginatum* invasion in France. However, to gain a deeper understanding of the invasion scenario, nuclear markers are necessary. Mitochondrial DNA is maternally inherited, lacks recombination, and exhibits a higher mutation rate compared with nuclear DNA, which can lead to an incomplete or potentially biased understanding of population history and gene flow [74]. SNPs were developed in 2024 by Hekimoğlu and Sağlam [75] and have enabled better understanding of the genetic diversity of *H. marginatum* in Turkey. Applying these markers to our samples could provide further insights into the genetic structuring of *H. marginatum* in France and the Mediterranean basin.

## Conclusions

To sum up, we have highlighted a complex history of *H. marginatum* introduction in France, with several populations that probably reflect distinct colonization events. Although there are doubts as to whether this is a recent introduction in France, we can nevertheless affirm that its abundance and range have expanded in recent years and will probably extend further north with ongoing climate change.

## Supplementary Information

Additional file 1. Table of individual specimens of *Hyalomma *analyzed during the study. Each row corresponds to a single individual, with specific details outlined in the columns, including specie, sampling location, stage, host, year, haplotype, cluster assignation, and Genbank number assignation.

Additional file 2. Nucleotide diversity of the two mitochondrial genes, cytochrome oxidase I and 12S rRNA. Ns = Number of sequences, S = Polymorphic sites, h = Number of haplotypes, k = Average number of nucleotide differences, Hd = Haplotypic diversity, π =Nucleotide diversity, D = Tajima’s D, Fs = Fu’s Fs. For Tajima’s D and Fu’s Fs, bold values are significant.

## Data Availability

Data are available within the manuscript and via GenBank (accessions numbers KX000609, KX000612, KX000615–KX000617, KX000619–KX000629, KX000632–KX000637, KX000639–KX000640, KX000642, KX000644–KX000650, MW366628–MW366633, MN885800, MT270686–MT270688, NC_056189, OQ263353, OQ263356–OQ263362, PQ885461–PQ885477, PQ894010–PQ894026, PQ897005, PQ899169, PQ899448–PQ899449, PQ899450, PQ899451–PQ899462, PQ900077–PQ900084, PQ902093–PQ902099, PV019529–PV020183, PV051478–PV052132, PV175379–PV175394).

## Abbreviations

AMOVA: Molecular analysis of variance
COI: Cytochrome oxidase I gene
DNA: Deoxyribonucleic acid
GHAD: Gard, Hérault, Ardèche & Drôme
PO-A: Pyrénées-Orientales & Aude
SNP: Single-nucleotide polymorphism
12S: 12S ribosomal RNA gene
